# Anticipatory neural dynamics of spatial-temporal orienting of attention in younger and older adults

**DOI:** 10.1016/j.neuroimage.2018.05.002

**Published:** 2018-09

**Authors:** Simone G. Heideman, Gustavo Rohenkohl, Joshua J. Chauvin, Clare E. Palmer, Freek van Ede, Anna C. Nobre

**Affiliations:** a Oxford Centre for Human Brain Activity, Wellcome Centre for Integrative Neuroimaging, Department of Psychiatry, University of Oxford, UK; b Brain and Cognition Lab, Department of Experimental Psychology, University of Oxford, UK

**Keywords:** Ageing, Anticipatory attention, Spatial orienting, Temporal orienting, Oscillations, MEG

## Abstract

Spatial and temporal expectations act synergistically to facilitate visual perception. In the current study, we sought to investigate the anticipatory oscillatory markers of combined spatial-temporal orienting and to test whether these decline with ageing. We examined anticipatory neural dynamics associated with joint spatial-temporal orienting of attention using magnetoencephalography (MEG) in both younger and older adults. Participants performed a cued covert spatial-temporal orienting task requiring the discrimination of a visual target. Cues indicated both where and when targets would appear. In both age groups, valid spatial-temporal cues significantly enhanced perceptual sensitivity and reduced reaction times. In the MEG data, the main effect of spatial orienting was the lateralised anticipatory modulation of posterior alpha and beta oscillations. In contrast to previous reports, this modulation was not attenuated in older adults; instead it was even more pronounced. The main effect of temporal orienting was a bilateral suppression of posterior alpha and beta oscillations. This effect was restricted to younger adults. Our results also revealed a striking interaction between anticipatory spatial and temporal orienting in the gamma-band (60–75 Hz). When considering both age groups separately, this effect was only clearly evident and only survived statistical evaluation in the older adults. Together, these observations provide several new insights into the neural dynamics supporting separate as well as combined effects of spatial and temporal orienting of attention, and suggest that different neural dynamics associated with attentional orienting appear differentially sensitive to ageing.

## Highlights

• Both younger and older adults benefit from cued spatial-temporal expectation.
• Alpha and beta lateralisation linked to spatial expectation is preserved and even strengthened in older adults.
• Temporal expectation modulates decreases in alpha oscillations in younger but not older adults.
• Combined spatial-temporal expectation increases contralateral gamma oscillations in older adults.
• Different anticipatory neural dynamics may be differentially sensitive to ageing.

## Graphical abstract

**Figure:**
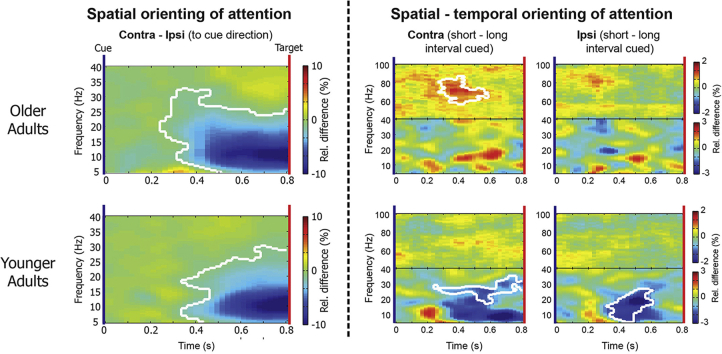

## Introduction

Our interactions with the world are adaptively shaped by our ability to anticipate relevant sensory events and orient our attention towards them. While such attentional orienting is classically studied with regard to how we orient our attention in space (i.e. “spatial orienting”; Posner et al., 1980), the question of how we orient our attention in time (i.e. “temporal orienting”) is equally relevant (Coull and Nobre, 1998; Nobre and Heideman, 2015; Nobre and van Ede, 2018). Moreover, studies point to a synergistic interaction between spatial and temporal orienting in vision, whereby the influence of temporal expectations is particularly pronounced when combined with spatial expectations (Doherty et al., 2005; Rohenkohl et al., 2014). This indicates that knowing when a relevant visual event is going to happen, in addition to where, allows you to prepare optimally to perceive and act upon this event.

In human electrophysiology, considerable progress has been made in understanding the anticipatory neural dynamics that support attentional orienting in space. The anticipatory attenuation of alpha (8–14 Hz) and beta (15–30 Hz) band oscillations in relevant sensory areas has been repeatedly reported, and proposed as a central mechanism for selectively regulating cortical receptivity based on spatial receptive fields (Worden et al., 2000; Thut et al., 2006; Wyart and Tallon-Baudry, 2008; Kelly et al., 2009; Jensen and Mazaheri, 2010; Snyder and Foxe, 2010; Gould et al., 2011; Haegens et al., 2011; van Ede et al., 2011). However, a number of outstanding questions remain unaddressed. First, whereas spatial and temporal orienting have each received ample investigation on their own, the neural dynamics that occur when they join forces remain largely unknown, despite good evidence for a synergistic relationship (Doherty et al., 2005; O'Reilly et al., 2008; Rohenkohl et al., 2014). Second, most previous studies investigating anticipatory neural dynamics in humans have focused on oscillations below 30 Hz. One study in non-human primates showed that temporal orienting of attention may additionally amplify higher-frequency gamma-band oscillations in visual cortex (Lima et al., 2011), which are suggested to reflect enhanced feedforward communication (Fries, 2015). It remains unclear whether visual gamma-band amplification can also be observed during temporal orienting in humans and, if so, whether such anticipatory gamma modulations interact with spatial orienting.

Furthermore, recent studies have started exploring how attentional orienting and its neuronal underpinnings change over the lifespan (see Zanto and Gazzaley, 2014, and Erel and Levy, 2016, for reviews). One study in particular argued for diminished benefits of temporal information with ageing (Zanto et al., 2011), showing declines in both behavioural and neural markers of temporal orienting (but see also Chauvin et al., 2016). Diminished anticipatory alpha-band modulations in older adults have also been noted for orienting attention in space (Deiber et al., 2013; Hong et al., 2015; but see also Leenders et al., 2016; Mok et al., 2016). Whether and how ageing also alters joint spatial-temporal orienting of attention, and the neural dynamics that support such orienting, remain unaddressed.

In the current study, we sought to investigate the anticipatory oscillatory markers of combined spatial-temporal orienting and to explore whether and how these decline with ageing. Younger and older adults engaged in a joint spatial-temporal orienting task, requiring a difficult perceptual discrimination following a spatial-temporal cue (cf. Rohenkohl et al., 2014), while we measured anticipatory neural dynamics using magnetoencephalography (MEG).

## Materials and methods

### Participants

Twenty younger adults (13 males, 7 Females; aged 24.1 ± 4.3 SD; age range 18–33) and twenty-one older adults (9 males, 12 females; aged 66.9 ± 4.5 SD; age range 61–76) completed the study. All but one participant were right-handed, according to self-report. All participants were healthy, had no history of neurological, psychiatric or vascular disease and had normal or corrected-to-normal vision. Data of three older adults had to be excluded due to large structural abnormalities visible on the structural magnetic resonance imaging (MRI) scan, technical problems during the recording and excessive artefacts in the MEG data, respectively. Data of two additional older adults had to be excluded because they performed below our cut-off threshold (<26) on the Montreal Cognitive Assessment (MoCA), which is indicative of a mild cognitive impairment (Nasreddine et al., 2005). A total of twenty younger adult datasets and sixteen older adult datasets (6 males, 10 females; aged 67.3 ± 5.0) remained for the analysis on which we report here. All participants gave informed consent, and the study was approved by the Central University Research Ethics Committee of the University of Oxford (MSD-IDREC-C1-2013-062 and MSD-IDREC-C1-2012-98) All participants were reimbursed for their time and travel expenses. The study was run in three (younger adults) or four (older adults) separate sessions (see 2.4.1 Overview of sessions) on separate days. Sessions were completed in the following order: a training visit, an MEG session, an MRI session, and a clinical and neuropsychological evaluation (older adults only).

### Clinical and neuropsychological evaluation

The older participants' data were collected as part of a larger “Cognitive Health in Ageing” research project. Therefore, in addition to the MEG study, older adults completed a set of demographic surveys, standardised clinical assessment questionnaires, and neuropsychological tasks to assess psychological health and basic cognitive function. This session took approximately 90 min. After the exclusion of two older adults who scored below 26 on the Montreal Cognitive Assessment (MoCA; see Nasreddine et al., 2005), all participants in the final analysis were within two standard deviations of normative values across all clinical assessment and neuropsychological tests.

### MEG and visual stimulation set-up

Whole-head MEG recordings were acquired at the Oxford Centre for Human Brain Activity using an Elekta NeuroMag (306 channel) MEG system. A magnetic Polhemus FastTrak 3D system (Vermont, United States) was used for head localisation. Relative positions of three anatomical landmarks (nasion, left and right auricular points) were measured in addition to relative positions of four head-position indicator coils.

MEG data were recorded in three (older adults) or four (younger adults) separate blocks of 12–15 min each, that were presented back to back with short breaks in between during which participants remained seated in the MEG chair. Data were sampled at 1000 Hz, and a bandpass filter between 0.03 and 300 Hz was applied during digitisation of the signal. ECG and horizontal and vertical EOG were recorded. In addition, eye movements were recorded with a video-based eye tracker with a sampling frequency of 500 Hz (EyeLink 1000, SR Research, Ontario, Canada). Fiber-optic response pads (made at BRU, Aalto University, Helsinki) were used to collect manual responses.

Stimuli were presented using MATLAB v.7.10 (The MathWorks, Inc., Natick, MA) and Psychtoolbox v.3.0 for MATLAB (Kleiner et al., 2007). The stimuli were back projected (Panasonic PT D7700E, Panasonic, Osaka Japan) on a 58 × 46 cm screen placed 120 cm in front of the participant, with a spatial resolution of 1280 × 1024 and a refresh rate of 60 Hz.

### Experimental procedure and stimuli

#### Overview of sessions

Both groups performed the same spatial-temporal orienting task, but there were some minor differences between the number of sessions, and the type and amount of data collected for younger and older adults. Younger adults took part in three sessions: a behavioural training session, an MEG session involving 800 trials of the spatiotemporal orienting task (see 2.4.2 MEG paradigm and procedure), and an MRI session in which an MRI T1 structural scan was obtained. Older adults took part in four sessions: a behavioural training visit, a second session involving MEG with 600 trials of the spatiotemporal orienting task and a resting-state recording (results of which will not be evaluated in the current manuscript), a third session including MRI T1 and T2 structural scans, a functional localiser scan, a resting-state scan and a diffusion tensor imaging scan (results of which will not be discussed in the current manuscript), and a fourth session involving an extensive clinical and neuropsychological evaluation (see 2.2 Clinical and neuropsychological evaluation).

#### MEG paradigm and procedure

**Fig. 1:**
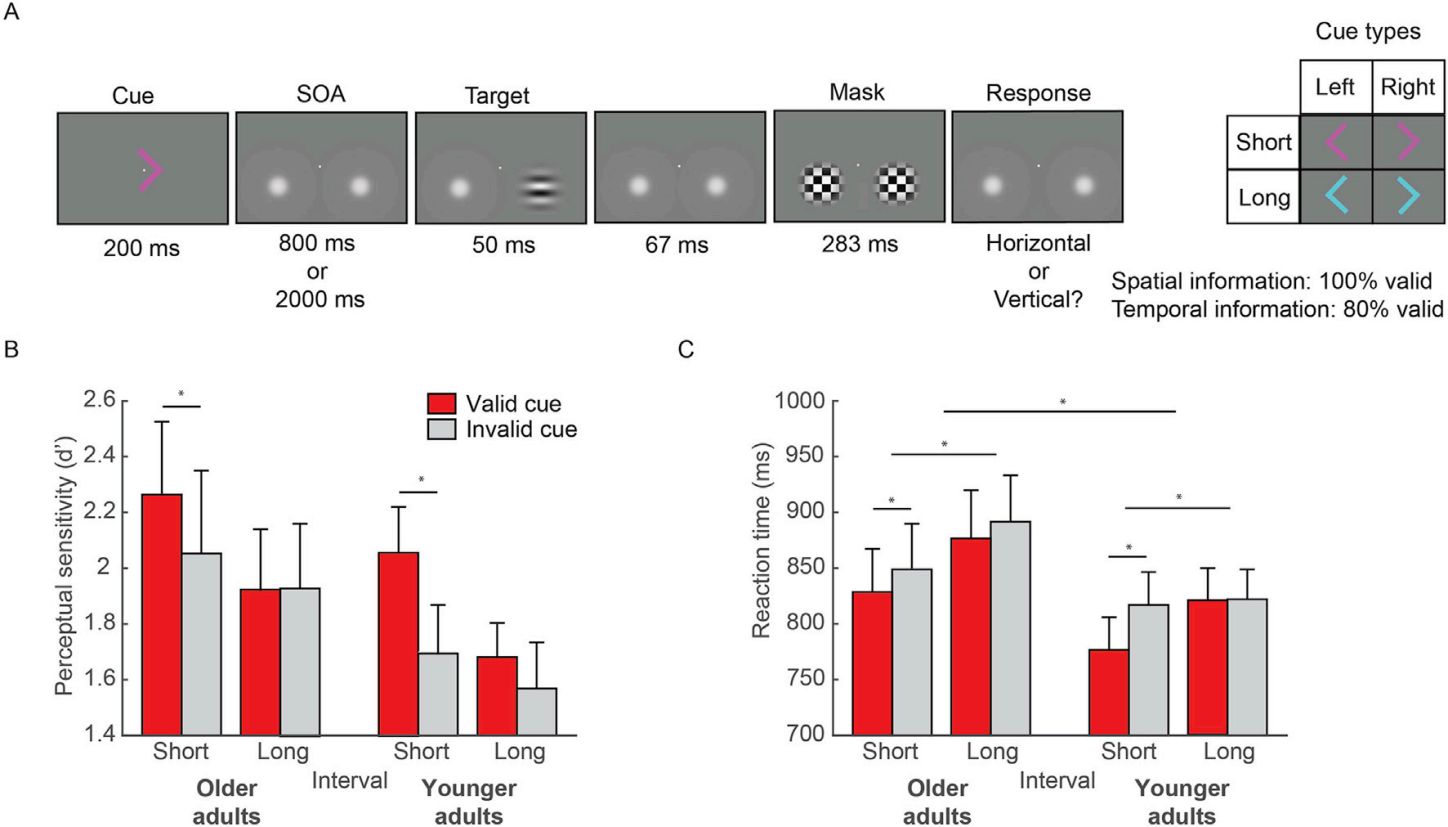
**Task and behavioural results.** (A) Behavioural task. Coloured arrow cues predicted where (bottom left or right, 100% valid) and when (after a short or long interval: 800 or 2000 ms, 80% valid) subsequent targets were likely to occur. Targets consisted of horizontally or vertically oriented Gabor patches followed by a backwards mask. Target discrimination performance was equated across participants by means of an adaptive staircase procedure using only valid spatial-temporal cues. Participants responded to the orientation of the Gabor grating by making left and right index finger responses. Note that stimuli are shown larger than actual size on screen for display purposes. Behavioural results are shown for (B) perceptual sensitivity (*d’*) and (C) reaction time (RT). Behavioural results are shown separately for older and younger adults, for each cue type (valid/invalid) and interval length (short/long). Error bars show standard error of measurement (SEM). Asterisks indicate statistically significant effects.

The experimental task is depicted in Fig. 1A. Participants had to discriminate the orientation (horizontal or vertical) of a peripherally presented target against a grey background while maintaining central fixation. Each trial started with a central fixation dot (diameter: 0.88° of visual angle) followed after 750–1200 ms by a foveally presented coloured arrow cue indicating where (left or right, 100% valid) and when (short cue/early: after 800 ms, or long cue/late: after 2000 ms, 80% valid) the upcoming target was likely to occur. Fully predictive spatial cues were used (100% valid) because Rohenkohl et al. (2014) showed that behavioural benefits of temporal expectation are restricted to targets occurring at attended spatial locations. Using only spatially valid cues enabled us to simplify the task and to focus on the neural dynamics of combined spatial-temporal attention.

Luminance pedestals positioned at 4.78° below the horizontal meridian and 3.38° from the vertical meridian (10% contrast) were presented throughout the experiment, indicating the two possible target positions. Cues were presented for 200 ms and consisted of blue or pink coloured arrows. Arrow direction indicated target location and the colour indicated when the target was most likely to appear. Colour-interval mappings were counterbalanced across participants. Both target locations and interval lengths were equiprobable and randomised across trials. Targets replaced the luminance pedestals for 50 ms. They were followed by a 283-ms backwards mask after a 117-ms target-mask SOA. Targets consisted of horizontally or vertically oriented Gabor patches with a diameter of 1.96° of visual angle and a spatial frequency of two cycles per degree of visual angle. The target contrast was adjusted individually using the adaptive staircase procedure (see MEG practice and staircase). Backward-mask stimuli were constructed by applying a Gaussian-vignette to the convolution of 100% square-wave gratings at the two possible target orientations. Responses were made with the left or right index finger, depending on target orientation. The response mapping was also counterbalanced across participants. A total of 800/600 (younger/older) trials were presented in randomised order (320/240 each for both the short and long valid temporal cues, and 80/60 each for the short and long invalid temporal cues), in three blocks of about 15 min each. Self-paced rest breaks were inserted every 20 trials.

#### MEG practice and staircase

The MEG session was preceded by a 90-min practice and staircase session, taking place on a separate day (Kaernbach, 1991; see also Rohenkohl et al., 2014 for a full description of the procedure that was followed). The practice involved 5 blocks in which the task became increasingly difficult, by decreasing the time of target presentation up to the presentation time of 50 ms. Once participants were practised and comfortable with the task, an adaptive psychophysical staircase procedure was run to estimate the threshold contrast for each participant. Task difficulty was adjusted by titrating the Gabor patch contrast until discrimination was performed at 75% accuracy. The calibration was run over 120 trials. After the calibration, each participant's performance was inspected to see if a 75% asymptote was reached. During the practice and staircase session all intervals were cued with 100% validity.

During the MEG session, which took place within one week after the practice session, participants performed a second practice consisting of 50 trials, using similar cue probabilities as used in the main MEG task. Participants were told that timing cues would now be correct in the majority of trials, but that they should still try to use the cued spatial and temporal information to predict where and when targets would occur. The MEG session, including set up and practice, took approximately 100 min for each participant.

### Behavioural analysis

The behavioural data were analysed using MATLAB, and statistics were performed in SPSS version 22 (IBM Corp. Armonk, NY). The MEG behavioural data were analysed with respect to perceptual sensitivity (*d’*) values and reaction times (RTs). Perceptual sensitivity was calculated according to the following formula:

d’ = z[percent correct horizontal] + z[percent correct vertical]

z[percent correct horizontal] and z[percent correct vertical] correspond to the inverse normal (z-score) transformations of the participants' proportions of correct responses to horizontal and vertical stimuli (see Rohenkohl et al., 2014). In cases where either of those values was equal to 0, the value was replaced with *0.5/N*; and if the value was equal to 1, it was replaced by *(N-0.5)/N*, where *N* reflects the number of trials with a horizontal or vertical stimulus for that condition, thereby penalising for conditions with fewer trials (i.e. conditions with invalid temporal cues). Trials with RTs smaller or larger than a participant's mean RT ± 3 times the standard deviation were excluded from the behavioural analysis. The behavioural results were analysed statistically using repeated-measures analysis of variance (ANOVA). Within subject effects were further evaluated using pairwise t-tests, while differences between both groups were evaluated using independent-samples t-tests.

### MEG analysis

#### Pre-processing and artefact rejection

MEG data analyses were performed using custom-written MATLAB code, the in-house OHBA Software Library (OSL) version 1.4 and Fieldtrip (Oostenveld et al., 2011). First, channels containing excessive noise were identified manually. Subsequently, spatiotemporal signal space separation (TSSS) and movement compensation were applied. Neuromag's MaxFilter software separates signals arising from inside and outside the helmet and thereby minimises extra-cranial noise (Taulu et al., 2004). The temporal extension of this signal space separation method removes interference from nearby sources (Taulu and Simola, 2006). In addition, MaxFilter compensates for the effects of head movement by using continuous head position measurements.

After using MaxFilter, the data were checked manually to ensure no problems occurred during the MaxFilter stage and down-sampled to 250 Hz. A 0.1-Hz high-pass filter was applied to the data to remove low-frequency drift. Because most datasets included periods with excessive noise recorded during rest-periods, the data were epoched before Independent Component Analysis (ICA) was performed, thereby ensuring that these periods with excessive noise would not negatively impact the ICA. ICA was used to reject artefacts associated with eye blinks, eye movements and heartbeat. All components were inspected manually and component time courses were compared with ECG and EOG time courses before removing them from the data. After ICA, the data were inspected manually to remove any remaining artefacts. Trials with eye blinks occurring during target presentation were excluded as well. On average 12% of trials were removed from the analysis. Trials with incorrect responses were not excluded from the MEG analysis because we were mainly interested in the anticipatory period before target appearance, i.e. before responses were being made. We note that when we repeated our MEG analysis using trials with correct responses only, results were comparable to the results reported here for all trials. Before analysis of high-frequency (gamma) oscillations, Fieldtrip's visual artefact rejection function showing summary statistics was run on data bandpass filtered between 40 and 100 Hz, to remove any remaining artefacts that were specific to the gamma-range.

#### Analysis of event-related fields for region of interest selection

**Fig. 2:**
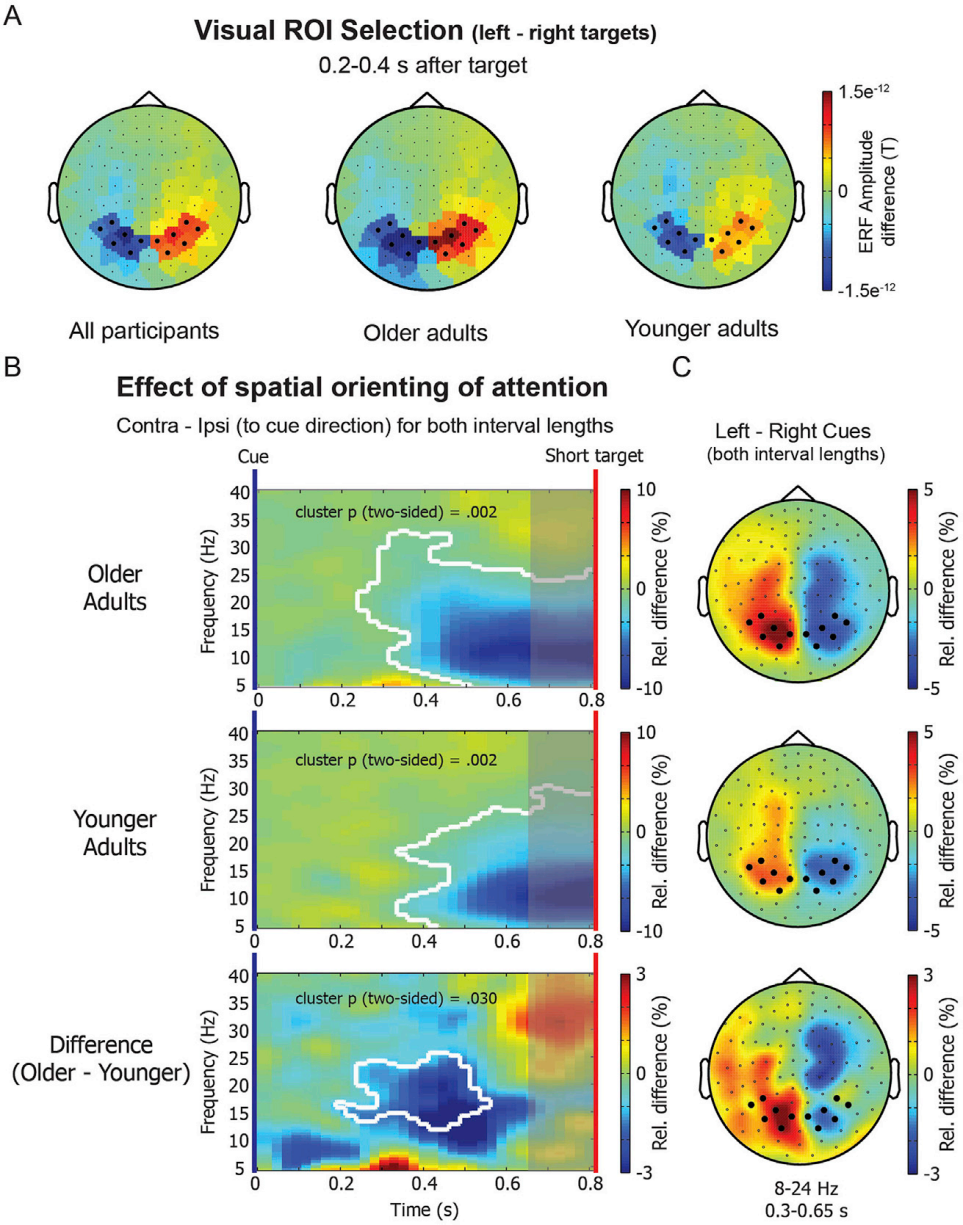
**MEG results for the effect of spatial orienting of attention.** (A) Topographies for the amplitude difference in the ERF 200–400 ms after target presentation in the left vs. right visual field (averaged over both short and long interval lengths). The six left and six right occipital-parietal channels that were used as ROIs throughout the subsequent MEG analysis are marked in black. Channels were selected based on visual inspection of the ERF grand average for all participants. Separate topographies for older and younger adults show that the largest ERF amplitude is found in the same channels for both groups. (B) Time-frequency representation (TFR) plots for older adults, younger adults and the difference between both groups (older-minus-younger), for contralateral ROI channels minus ipsilateral ROI channels (to the direction of the cue), averaged over both cue-target interval lengths. Results are shown for the short interval (0–800 ms) only. The colour scale indicates a relative increase or decrease. Significant clusters are outlined in white. The shaded area indicates a time period that cannot be considered purely anticipatory, because target-evoked activity might bleed in. (C) Topographies for the left-minus-right cue contrast (averaged over both interval lengths), separately for older adults, younger adults and the difference between both groups (older-minus-younger). Topographies are averaged over frequencies between 8 and 24 Hz and for a time window between 300 and 650 ms. Black dots represent the contralateral and ipsilateral visual ROI channels.

To avoid overlap with the main time period of interest (0–800 ms after cue onset) or main measure of interest (time-frequency data between 4 and 100 Hz), visual ROIs were selected based on target-evoked ERFs. ERFs were calculated separately for all four combinations of target location and interval (left-short, right-short, left-long and right-long). ERFs were baseline-corrected with the average of the window 100 ms before target presentation (700–800 ms after the cue for targets appearing after the short interval and 1900–2000 ms for targets appearing after the long interval). Data for the planar gradiometer pairs were combined, resulting in a 102-channel combined planar gradiometer map in sensor space. Subsequently, ERFs for short and long targets were aligned at target presentation, and averaged separately for left and right targets. Finally, a left-right difference ERF was calculated for each participant and averaged across all participants. This difference ERF topography was plotted 200–400 ms after target presentation, where the difference was maximal. Based on the topography (see Fig. 2) six (symmetric) channel pairs were selected on the left (MEG1632 + 1633; MEG1642 + 1643; MEG 1912 + 1913; MEG 1922 + 1923; MEG 1942 + 1943; MEG 2042 + 2043) and on the right (MEG 2032 + 2033; MEG2312 + 2313; MEG2322 + 2323; MEG2342 + 2343; MEG2432 + 2433; MEG2442 + 2443). Channel pairs were selected based on the grand average of all participants, but are also shown separately for younger and older adults in Fig. 2 to demonstrate that channels with maximum lateralised ERFs were the same for both groups.

#### Time-frequency analysis

Time-frequency analysis was performed using a short-time Fourier Transform, separately for lower (4–40 Hz) and higher (40–100 Hz) frequencies. For both analyses, we used a fixed sliding time window of 300 ms that was advanced over the data in steps of 25 ms. For the lower frequencies, we used a Hanning taper, and estimated power between 4 and 40 Hz, in 0.5 Hz steps. For the higher frequencies, we used a multi-taper approach (Percival and Walden, 1993) using ± 8 Hz spectral smoothing, and estimated frequencies between 40 and 100 Hz, in steps of 1 Hz.

For both lower and higher frequencies, the power spectra were averaged separately over trials for each cue condition. The power time series in the planar gradiometer pairs were then combined (Cartesian sum), resulting in a 102-channel combined planar gradiometer map in sensor space. All relevant time-frequency contrasts were computed as the average of the selected ROI channels (see 2.6.2 Analysis of event-related fields for region of interest selection) and subsequently averaged for ROIs contralateral and ipsilateral to the cue direction. All contrasts were computed as relative contrasts. For example, the contrast between short vs. long cues was calculated as follows (separately for contralateral and ipsilateral channels):

((Short Cue - Long Cue)/(Short Cue + Long Cue)) x 100

Contrasts of interest were evaluated during the short interval (0–800 ms). This is the time period during which temporal expectations differ between cueing conditions. After the first interval passes, it becomes evident that the target will appear after a long interval irrespective of the initial cue, so temporal cueing benefits dissipate as it is now. Differences between conditions were investigated by means of cluster-based non-parametric permutation testing in Fieldtrip using 1000 permutations with a cluster alpha of 0.05. This approach circumvents the multiple-comparisons problem (Maris and Oostenveld, 2007). The resulting p-values were multiplied by a factor of 2, to reflect two-sided statistical evaluation of our results.

### Analysis of eye-tracking data

Eye-tracking data were absent or not usable for six younger adults and two older adults. Due to the way the recording was set up, we always got an eye tracker signal, even when the eye tracker was not pointed at the eyes, or when the eye tracker partially or fully “lost” the eye during the recording. The eight non-usable recordings either had a flat signal with lots of 50Hz noise and some drift (6 participants), or a very unstable signal (2 participants). We believe the flat recordings reflect missing data, while the unstable signal reflects the eye tracker “losing” the eye, e.g. because the participant was wearing thick glasses that made it difficult to record, or the eye tracker not being optimally positioned in the first place. Therefore, data from the remaining fourteen younger adults and fourteen older adults were included in the eye-tracking analysis. Eye-tracking analysis was performed on the x-position eye-tracker data (i.e. gaze in the horizontal plane) during the short interval, recorded in Volt as a separate channel on the MEG system. All trials that were rejected during pre-processing (see 2.6.1 Pre-processing and artefact rejection) were rejected from the eye-tracking analysis as well. Participant averages were calculated separately for all four combinations of cued target location and interval (left-short, right-short, left-long and right-long) and averaged separately for younger and older adults. Data were baselined using the 500-ms window before cue presentation.

### Post-hoc analysis of correlation between MEG data and behaviour

In a series of post-hoc analyses we investigated the link between our MEG results and behaviour. First, we conducted a logistic regression between accuracy and average MEG power between 300 and 650 ms after short cue onset (valid trials only), separately for the alpha (8–14 Hz), beta (15–28 Hz) and gamma (60–75 Hz) bands. Second, we computed the trial-wise correlation between power in the same time-frequency windows and RT (again, for validly cued short trials only). The results of these analyses are presented in Supplementary Figure S1 and Tables S1 and S2. Finally, we performed across-participant correlations between the power difference following short vs. long cues (averaged across a time window between 300 and 650 ms, separately for alpha, beta and gamma) and the behavioural validity effect (behaviour for short valid minus long invalid cues) for both RT and accuracy. These results are shown in Supplementary Figure S2 and Tables S3 and S4. Given our limited number of trials and participants, and the large number of statistical tests that we performed, we believe that any of these findings should be interpreted with caution.

## Results

We measured magnetoencephalographic brain activity in healthy human volunteers, while they engaged in a joint spatial-temporal orienting task (Fig. 1A). Coloured arrow cues indicated where (left or right, 100% valid) and when (after 800ms/early or after 2000ms/late, 80% valid) upcoming visual targets were most likely to be presented, and participants indicated the orientation (horizontal or vertical) of this target by making a left or a right button press.

### Behavioural results

Behavioural results for perceptual sensitivity (*d’*) and reaction time (RT) are shown in Fig. 1B and C. To investigate the difference between valid and invalid temporal cues (spatial cues were always valid), i.e. the “temporal validity effect”, a repeated-measures analysis of variance (ANOVA) was performed, with the within-subject factors cued interval (short or long: 800 or 2000 ms) and validity (valid vs invalid) and the between-subjects factor age group. For *d’*, a main effect of cued interval (F(1,34) = 9.33, p = .004, partial η^2^ = 0.22) and a main effect of validity (F(1,34) = 7.39, p = .01, partial η^2^ = 0.18) were found. The interaction between cued interval and validity showed a trend, but did not reach significance (F(1,34) = 3.51, p = .07, partial η^2^ = 0.09). When, however, testing the validity effect separately for both intervals, we could confirm a significant benefit of valid temporal cues at the short, but not at the long interval (short: t(35) = 2.87, p = .007, Cohen's d = 0.48; long: t(35) = .87, p = .39, Cohen's d = 0.14), in line with a number of previous studies (see e.g. Coull and Nobre, 1998; Miniussi et al., 1999; Nobre, 2010; Rohenkohl et al., 2014). No main effect of group (F(1,34) = 1.36, p = .25, partial η^2^ = 0.04) or three-way interaction with group (F(1,34) = .02, p = .90, partial η^2^ = 0.00) was present.

For RT, we found a main effect of cued interval (F(1,34) = 23.60, p < .0001, partial η^2^ = 0.41), a main effect of validity (F(1,34) = 19.77, p < .0001, partial η^2^ = 0.37) and an interaction between cued interval and validity (F(1,34) = 7.27, p = .011, partial η^2^ = 0.18). There was no main effect of group (F (1,34) = 1.36, p = .252, partial η^2^ = 0.04), but there was a three-way interaction between cued interval, validity and group (F(1,34) = 4.166, p = .049, partial η^2^ = 0.11). Pairwise t-tests revealed that for both older and younger adults a validity effect was again present for the short (older: t(15) = 2.54, p = .023, Cohen's d = −0.63; younger: t(19) = 6.13, p < .0001, Cohen's d = −1.37), but not for the long interval (older: t(15) = 1.72, p = .105, Cohen's d = −0.43; younger: t(19) = 0.17, p = .87, Cohen's d = −0.04). An independent samples *t*-test showed that the magnitude of the temporal validity effect for short cues was larger in younger, than in older adults (t(34) = 2.09, p = .045, Cohen's d = 0.70).

To summarise, for both *d’* and RT, performance was better following valid, compared to invalid temporal cues. These performance benefits were present following short, but not following long intervals. Based on these findings, we thus expected the largest neural differences due to temporal expectations (i.e., in the contrast between short and long cues) to occur in the short interval, and therefore focused on this interval in the MEG analysis on which we report below.

### MEG results

#### Visual region-of-interest selection

Fig. 2A shows the regions-of-interest (ROIs) that were used to extract left and right (and thus contra- and ipsilateral) visual activity. The left and right occipital-parietal ROIs were selected after visual inspection of event-related field (ERF) topographies for the difference between left and right targets in the interval between 200 and 400 ms post-target. ROI selection was performed on the grand average across all participants. The same sensors would have been selected based on either the younger or older adult averages in isolation (see Fig. 2A).

#### Spatial orienting effects in younger and older adults

Fig. 2B zooms in on the spatial orienting effect by showing the difference in power (across time and frequency) between trials in which the cued side was contralateral vs. ipsilateral to the selected ROIs (collapsed across both ROIs and short and long temporal cues). To stay consistent with all further analysis, we focused exclusively on the early anticipatory interval (0–800 ms). Results are shown separately for older (top plot) and younger (middle plot) adults, as well as the difference between these groups (bottom plot). Significant clusters (outlined in white, corrected for multiple comparisons across time and frequency; Maris and Oostenveld, 2007) were found in these time-frequency plots for the anticipatory lateralisation for both older and younger adults (older adults: cluster p (two-sided) = .002; younger adults: cluster p (two-sided) = .002), starting from approximately 300 ms after cue presentation, in a frequency band that spanned roughly from 5 to 25 Hz (i.e. encompassing both alpha and beta bands). These results are in line with a relative reduction of alpha and beta power contralateral to the locus of attention and replicate well known patterns previously reported for spatial orienting of attention in younger adults (Worden et al., 2000; Thut et al., 2006; Kelly et al., 2009; Gould et al., 2011; Händel et al., 2011; Deiber et al., 2013) and additionally demonstrate that this pattern is similar for older adults (see also Leenders et al., 2016). In fact, the bottom plot shows that, in our data, this lateralisation was even stronger in older than in younger adults (cluster p (two-sided) = .030), with a significant group-effect cluster that localises to the higher portion within this frequency range. Note that in this analysis we collapsed across both cued intervals, to first establish a pure spatial orienting effect. However, we note that results look nearly identical for short and long cues. We also note that although our statistical analyses presented here and below were performed on the full 800-ms window, of which the last 150 ms might not be purely anticipatory (provided our sliding time window of 300 ms; see 2.6.3 Time-frequency analysis). Crucially, however, for all reported analyses, equivalent results were obtained when only evaluating the first 650 ms of this window (Figures S3 and S4).

Associated topographies of the spatial lateralisation effect (i.e. left vs. right cues) are shown in Fig. 2C, averaged over frequencies between 8 and 24 Hz and between 300 and 650 ms post-cue (i.e. the time period and frequency range that closely resemble the observed clusters). Topographies are plotted up to 650 ms to reflect only the interval that is purely anticipatory. Note that topographies look highly similar in both groups and that the group difference (stronger lateralisation in older adults) also appears to originate, at least in large part, from the selected posterior channels.

#### Bilateral desynchronization in younger but not older adults during temporal orienting

**Fig. 3:**
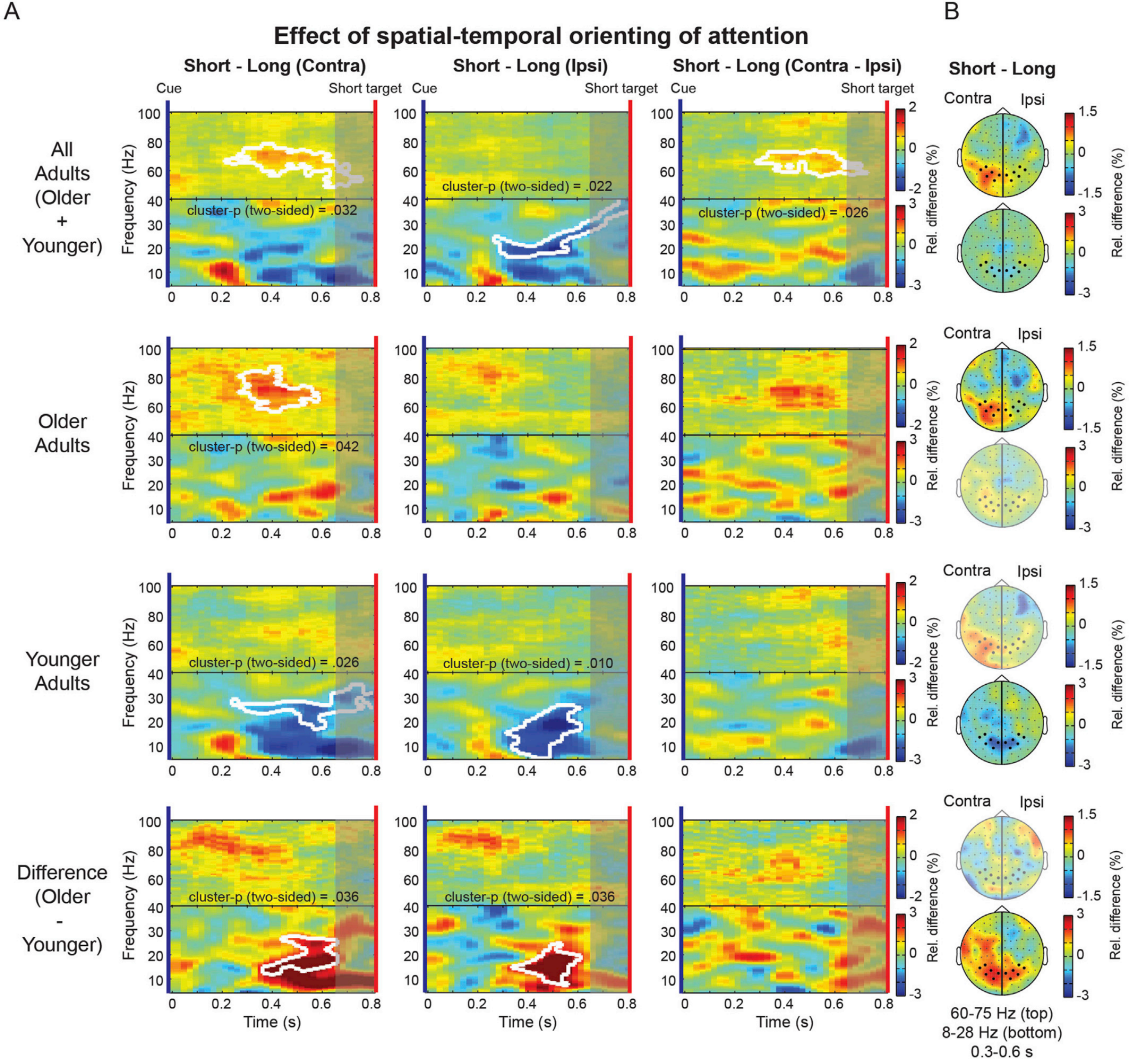
**MEG results for the effect of spatial-temporal orienting of attention**. (A) Time-frequency representation (TFR) plots showing the short-minus-long cue contrast for all participants (older-plus-younger), older adults, younger adults and the difference between groups (older-minus-younger), separately for ROI channels contralateral (first column) and ipsilateral (second column) to the cue direction and for the contra-minus-ipsi contrast (third column). Results are shown for the short interval (0–800 ms) only. The colour scale indicates a relative increase or decrease. Significant clusters after nonparametric cluster-based permutation testing are outlined in white. The shaded area indicates a time period that cannot be considered purely anticipatory, because target-evoked activity might bleed in. (B) Topographies for the same short-minus-long contrast, for contralateral (shown on left) and ipsilateral (shown on right) channels. Topographies display activity averaged over frequencies between 60 and 75 Hz (top) and 8–28 Hz (bottom) and for a time window between 300 and 600 ms after cue presentation. Black dots represent the contralateral and ipsilateral visual ROI channels.

We next separated the trials further by also separating “short” and “long” temporal cues. We again focused on the short interval (0–800 ms post-cue). Based on the behavioural data, it is expected that, in this short interval, anticipation of the target is stronger when the trial had started with a “short” cue, compared to when it had started with a “long” cue. Fig. 3A shows the difference between short- and long-cue trials in this short interval, separately for contralateral and ipsilateral ROIs, as well as for their difference (i.e. contra (short – long) minus ipsi (short – long); i.e. the spatial-temporal interaction effect).

Focusing on the lower frequencies (bottom part of each plot in Fig. 3A) for the combined groups (top row), we observed an effect of temporal orienting of attention (i.e. short – long cues) for the ipsilateral ROI (cluster p (two-sided) = .022), with a contralateral effect going in the same direction, but not reaching significance. When comparing the effect for both groups, it becomes clear that this effect of temporal orienting was present in younger adults only, now reaching significance in both contralateral (cluster p (two-sided) = .026) and ipsilateral (cluster p (two-sided) = .01) ROIs. Younger adults showed a bilateral power decrease (desynchronisation), while no significant effects were found in older adults. This difference between older and younger adults was also reflected in the older-minus-younger contrast over both contralateral (cluster p (two-sided) = .036) and ipsilateral (cluster p (two-sided) = .036) ROIs. These effects are also visible in the topographies for the short-minus-long cue contrast, as depicted in Fig. 3B, averaged for frequencies between 8 and 28 Hz (resembling the observed clusters). The bilateral attenuation in low-frequencies found in younger adults is clearly reflected here, while this pattern again appeared to be absent in older adults. We also noted that the alpha-band lateralisation in the younger adults appeared slightly more pronounced following short compared to long cues (or, analogously a stronger temporal modulation contralateral compared to ipsilateral – i.e., a spatial-temporal interaction), starting from 600 ms. However, since this effect overlapped with the window which could be contaminated by target-evoked activity, we cannot be certain if this effect is truly anticipatory. Much of the effect of temporal orienting in younger adults in this study was therefore largely independent of concurrent spatial expectations, and may not be key to explaining joint spatial-temporal orienting effects. Indeed, Fig. 3 makes it clear that even though there was a strong lateralisation with spatial orienting in both groups (Fig. 2B), the strength of this lateralisation was not different between short and long cues. It therefore did not appear in Fig. 3, where we focus on differences between short and long cues (i.e. effects of temporal, and spatial-temporal orienting).

#### Spatial-temporal orienting in the gamma band in older adults

Interestingly however, when focusing on the higher frequencies (Fig. 3A upper panels), we observed, across the whole group, a temporal attention modulation that appears specific in time as well as in space. We found significant short-minus-long clusters both for the ROI contralateral to the cue direction (cluster p (two-sided) = .032) and for the contra-minus-ipsi difference (cluster p (two-sided) = .026), indicating larger gamma power in this interval following short, compared to long cues. When investigating this effect in both groups separately, this effect appeared largely exclusive to older adults. A significant cluster was found for this age group, for the ROI contralateral to the cue direction (cluster p (two-sided) = .042). The topographies (Fig. 3B, upper topography plots) confirmed that the pattern of gamma amplification occurred most strongly in contralateral posterior channels (again in line with an interaction between temporal and spatial orienting). In younger adults no significant clusters were observed, and anticipatory gamma modulations were either much weaker, noisier, or not present at all. Nevertheless, while the time-frequency plots revealed neither clear signs of anticipatory gamma modulations nor significant high-frequency clusters in this age group, it is still noteworthy that, when considering the topographies, the data in the younger adults appear to hint at a possible modulation with similar spatial-temporal nature as observed in the full group as well as in the older adults.

In a series of post-hoc analyses, we investigated the relationship between the effects in our MEG data and behavioural performance (both across trials and across participants). We could not find compelling effects, which is likely due to relatively low numbers of trials and participants. For transparency, results of these analyses are presented in supplemental Figures S1 and S2 and supplemental Tables S1-S4.

### Gaze analysis

**Fig. 4:**
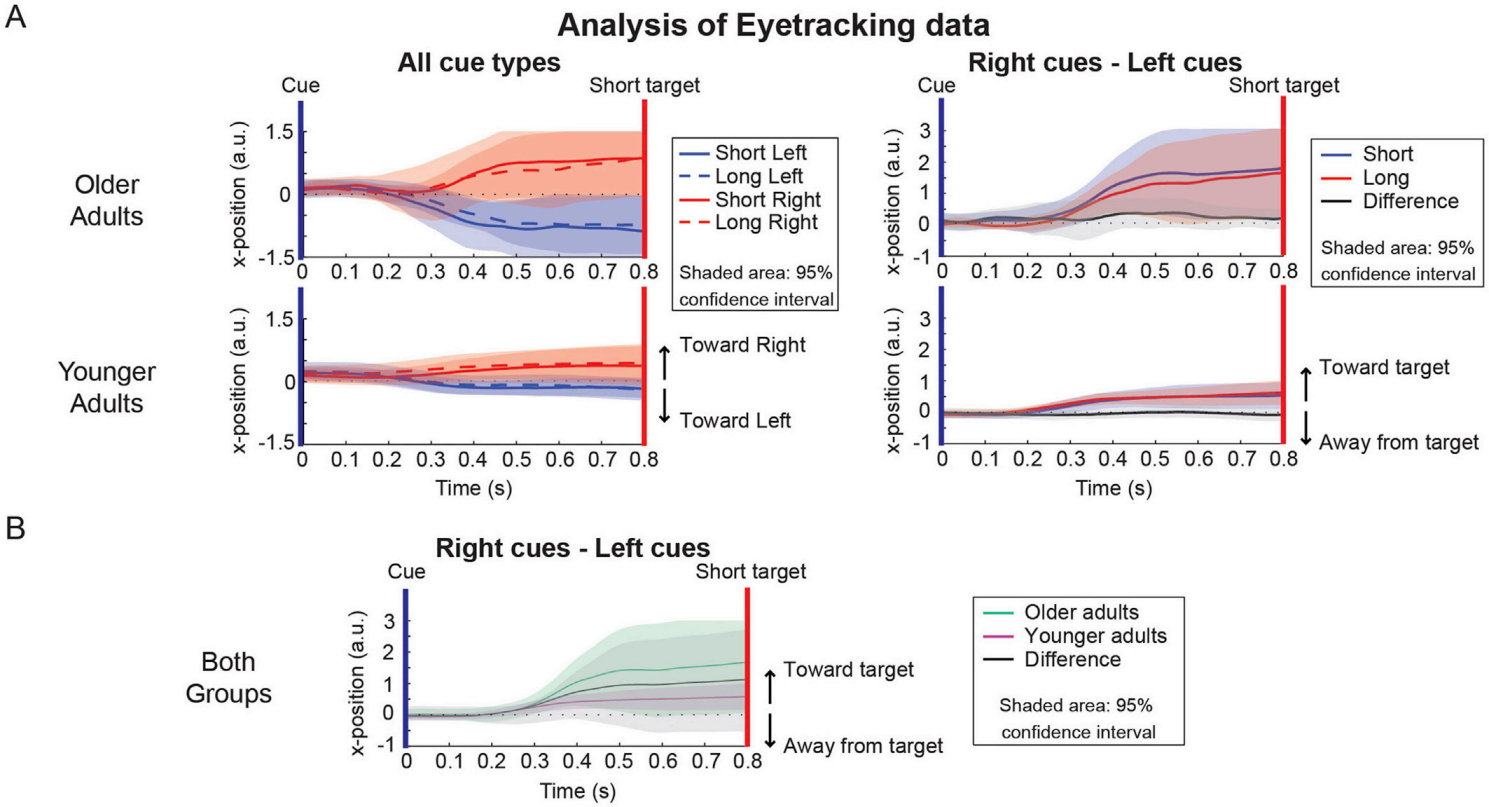
**Analysis of eye-tracking data.** (A) Data are shown for older (top) and younger (bottom) adults separately. The left side shows data for all cue types. The y-axis shows the x-position of the eye tracker, with 0 being completely centre. The right side shows data for the size of the difference in x position between left and right cues, for both the short and the long interval. All data are plotted with the 95% confidence interval (shaded area). Note that the (grey) confidence interval of the difference line overlaps with the zero crossing, indicating that there was no significant difference in the size of horizontal eye movements between short and long cues. (B) Results for older and younger participants, averaged across short and long cues, for the difference between left and right cues. Note that the (grey) confidence interval of the difference line overlaps with the zero crossing, indicating that there was no significant difference in the magnitude of horizontal eye movements between older and younger adults.

We evaluated the eye-tracking data to investigate whether the spatial-temporal orienting effects may have been driven by gaze differences between conditions (or groups), i.e. by more gaze shifts in the direction of the cued target location after short, compared to long cues. The left graph in Fig. 4A shows the average x-position over time for both older (top) and younger (bottom) adults over the course of the short interval, for all cue conditions, with the shaded area reflecting the 95% confidence interval. The graph on the right shows the average left-minus-right cue difference over time, presented separately for short and long cues. Although these graphs show a tendency for both younger and older adults to gaze toward the anticipated target location, this tendency was highly stereotyped and showed no difference between short and long cues and therefore did not account for any of the reported spatial-temporal orienting effects (indeed, the 95% confidence interval for this difference always overlapped with the zero line; see Fig. 4B, right panel). As an additional control analysis, we removed the spatial gaze bias through stratification (resulting in an average gaze bias away from the cued side) and recomputed our time-frequency plots with the remaining trials (as in van Ede et al., 2017). Time-frequency plots before and after removal were virtually indistinguishable (Figure S5), suggesting that also the spatial lateralisation effects were unlikely driven purely by biases in gaze.

Returning to the spatial gaze shift, we were concerned that the stronger lower frequency lateralisation observed with spatial cueing in the older adults may have been driven by a larger spatial gaze bias in this group. We therefore decided also to evaluate the difference in spatial gaze bias between older and younger adults (see Fig. 4B). As before, although there seems to be an optical difference between younger and older adults, this difference never reached statistical significance (i.e. the confidence interval of this difference overlaps with 0 for all tested time points). Furthermore, when reverting the eye tracker traces in both groups (as described above; see also Figure S5), the group difference too remained highly similar, suggesting that also the observed group difference was unlikely attributed to potential differences in gaze biases between the groups.

## Discussion

We investigated anticipatory neural dynamics related to spatial-temporal orienting of attention using MEG, in both younger and older adults. In both age groups, we replicated well known effects of spatial orienting by showing lateralisation of posterior alpha and beta oscillatory power (Worden et al., 2000; Thut et al., 2006; Wyart and Tallon-Baudry, 2008; Kelly et al., 2009; Jensen and Mazaheri, 2010; Snyder and Foxe, 2010; Gould et al., 2011; Haegens et al., 2011; van Ede et al., 2011). In contrast to several previous reports (discussed below), however, this spatial modulation was not attenuated but, instead, enhanced in the older adults. In contrast, a pure temporal orienting effect involving a suppression of alpha and beta oscillations was only observed in younger adults. Finally, the joint forces of spatial and temporal expectations resulted in a temporally and spatially specific amplification of anticipatory gamma-band oscillations. When we considered this effect for both groups separately, this effect was only compelling in the older adults.

### Neurophysiology of spatial-temporal orienting

Spatial and temporal orienting of attention are typically studied in isolation and have each been associated with anticipatory attenuation of alpha and beta oscillations (spatial: Worden et al., 2000; thut et al. ,2006, Wyart and Tallon-Baudry, 2008; Kelly et al., 2009; Jensen and Mazaheri, 2010; Snyder and Foxe, 2010; Gould et al., 2011; Haegens et al., 2011; van Ede et al., 2011; temporal: Rohenkohl and Nobre, 2011; van Ede et al., 2011; Zanto et al., 2011). Such attenuations are generally attributed to an anticipatory increase in neuronal excitability (Pfurtscheller and Lopes da Silva, 1999; Jensen and Mazaheri, 2010; Haegens et al., 2011; Jenkinson and Brown, 2011), and have also been proposed to relate to feedback processes (Buffalo et al., 2011; Bastos et al., 2015). In addition to replicating such anticipatory patterns by and large, one particular aim of the current study was to look at combined effects of both forms of attentional orienting. Rohenkohl et al. (2014) showed a strong synergy at the behavioural level between temporal and spatial orienting (see also e.g. Doherty et al., 2005; O'Reilly et al., 2008). Such a synergy would be expected to be supported by anticipatory neural modulations that are both spatially and temporally specific. To our surprise, modulations in the alpha and beta-bands did not show such clear spatial-temporal interactions in the current study (in contrast to van Ede et al., 2011 and Heideman et al., 2017) – although our results did show a robust bilateral attenuation during temporal orienting in younger adults (which was also observed in van Ede et al., 2011, as well as in Rohenkohl and Nobre,2011). Instead, at least in older adults we found a complementary anticipatory modulation in the gamma-band that was both spatially and temporally specific, which we discuss further below.

### Anticipatory gamma dynamics

In older adults, an interaction between spatial and temporal orienting was found in the gamma band, expressed in an anticipatory amplification of 60–75 Hz oscillations in contralateral parietal channels, with stronger gamma power in the short interval when targets were expected to occur early. In younger adults the topographies hinted at a possibly similar (albeit weaker) modulation.

Anticipatory modulations of gamma-band activity related to temporal orienting have previously been reported by Lima et al. (2011) in a study with non-human primates. They showed anticipatory enhancement of gamma in primary visual cortex (V1) following temporal cues when monkeys expected a task-relevant fixation cross change in time. In contrast to our study, however, Lima and colleagues investigated temporal gamma modulations without varying spatial expectations. The spatial specificity of the effect could not, therefore, be evaluated in their study.

In humans, reports of anticipatory gamma modulations are rare (but see van Ede et al., 2014) and such modulations have not previously been reported in the context in which both temporal and spatial orienting are present concurrently. van Ede et al. (2014) focused on spatial attention in the somatosensory modality in younger adults and found small but significant lateralised gamma-band power increase over somatosensory areas contralateral to the cued side, which was similar in range, although much weaker, than gamma modulations during sustained stimulus processing. Here we report a similar effect in human visual cortex and, moreover, show that this is also temporally specific.

There are at least three possible reasons why most previous studies may not have found anticipatory modulations of gamma-band activity. First, a constant, driving, visual input might be necessary for detectable gamma-band modulations to occur, like the continuously visible luminance pedestals in the current study, or the constant grating in Lima et al. (2011). Such an effect may reflect the pre-target instantiation (or amplification) of a feedforward communication channel (in line with e. g Fries, 2009, 2015; Bastos et al., 2015; possibly enabled through the driving input of the pedestal), such that when the target comes in, it can efficiently be broadcasted to downstream areas. Second, previous studies simply might not have looked at higher frequencies, either because they focused on well described modulations of lower alpha and beta-band oscillations, or because they were based on electroencephalography (EEG) instead of MEG. Third, it might be the case that anticipatory gamma modulations are strongest when information about both space and time can be used to guide attention to relevant perceptual events, while most previous studies focused on either spatial or temporal orienting alone. Moreover, given that the spatial-temporal gamma modulations were only clearly evident in the older adults, such a modulation may be missed or be absent in studies that focus exclusively on younger adults. Clearly, further research is necessary to elucidate the exact conditions under which anticipatory effects of spatial-temporal orienting occur in the in gamma band. In future studies it will be important to investigate if these spatial-temporal anticipatory gamma modulations are truly limited to older adults (and therefore might reflect some form of compensatory activity with ageing), or whether these modulations can also be reliably revealed in younger adults, for example with larger sample sizes.

Our findings highlight that when gamma oscillations are amplified, alpha and beta oscillations do not always concurrently decrease in power. This is also in line with research by Scheeringa et al. ,2011, who showed in a combined EEG-fMRI study that gamma-band oscillations correlated with the BOLD signal independently of alpha- and beta-band oscillations. Other studies also suggest that alpha/beta and gamma modulations need not always go hand in hand, but may sometimes occur independently of each other (e.g. Bauer et al., 2014; van Ede et al., 2014; Shaw et al., 2017).

### Spatial-temporal orienting and anticipatory brain dynamics in ageing

Older adults are usually found to maintain the ability to orient attention to specific spatial locations to improve performance (Zanto and Gazzaley, 2014; Erel and Levy, 2016). However, anticipatory alpha modulations with such attentional orienting are sometimes found to be reduced (Deiber et al., 2013), and some reports suggest that attentional deployment in older adults might not be reliant on alpha power lateralisation at all (Hong et al., 2015).

Contrary to these findings, and more in line with the current results, a recent study using a lateralised working-memory task (Leenders et al., 2016) showed similar alpha lateralisation in younger and older adults during the attentional cue period preceding a to-be-remembered sample array. Likewise, Mok et al. (2016) recently found largely preserved alpha and beta lateralisation in older adults with a different working-memory paradigm that used retro-cues to orient attention to specific locations of items maintained in working memory. These studies show that, under some circumstances, older adults show well preserved lateralised low-frequency (alpha and beta) activity when directing their attention to one cued hemifield, at least for short periods of time. The current study suggests that in some cases this lateralisation might be even stronger in older adults, at least in the beta band. There are at least two possible explanations for this effect. While this may reflect a true amplification of this modulation in older adults, an alternative explanation is that this modulation is more diffuse in its spectral distribution in older adults – consistent with the group difference peaking in the beta band range (see Fig. 2).

The degree to which the benefits of temporal orienting are affected by ageing, has also been a matter of recent debate. Zanto et al. (2011) have shown that beneficial effects of temporal information may decline with ageing, using a variety of cued temporal-orienting tasks. Both behavioural performance and anticipatory EEG modulations were affected, suggesting that the effects of temporal cues diminish with ageing. Contrary to these findings, Chauvin et al. (2016) recently showed preserved temporal orienting abilities in healthy ageing using speeded and non-speeded behavioural tasks. Both Zanto et al. (2011) and Chauvin et al. (2016) used central targets and central cues. However, as shown by Rohenkohl et al. (2014), the strongest effect of temporal expectations can be found when both space and time are relevant and interact, and the task to be performed is perceptually challenging. In the current study, older adults were clearly able to benefit behaviourally from temporal cues. Moreover, when considering the joint spatial-temporal orienting effect (i.e. the gamma band modulation), this effect appeared restricted to older adults. It will be interesting to address in future research whether, for example, synergistic behavioural effects observed previously in young adults (Rohenkohl et al., 2014) are equally well preserved in ageing (as our MEG data suggest).

Our data have revealed that different neural dynamics associated with attentional orienting may be differentially sensitive to ageing, with some effects appearing exclusively in younger adults (bilateral alpha modulation with temporal orienting), whereas others appear most clearly or even exclusively in older adults (spatial-temporal gamma modulation). In future work it will be interesting to assess to what extent these changes with age must be attributed to changes in physiology, different strategies, or compensatory processes.

## Conclusions

The main insights from the current work are threefold. First, when visual spatial and temporal attention join forces, we find certain anticipatory neural dynamics (i.e. in the gamma band) that are sensitive to joint spatial-temporal expectations, while others (i.e. in the alpha and beta bands) are mainly sensitive to spatial and temporal expectations by themselves, irrespective of concurrent expectations in the other dimension. Second, we demonstrate the involvement of anticipatory neural dynamics in the gamma band in older humans and show that it is possible to study this non-invasively using extracranial MEG recordings. Finally, we find that different anticipatory neural dynamics are not always diminished, but sometimes are even more pronounced in older adults.
