# Supplementary material for: Anticipatory neural dynamics of spatial-temporal orienting of attention in younger and older adults
Source: Neuroimage. 2018 Sep;178:46–56. doi: 10.1016/j.neuroimage.2018.05.002 (PMC6057272; doi:10.1016/j.neuroimage.2018.05.002)
Supplement: Supplementary_material [file mmc1.pdf]

Supplementary Material

Anticipatory neural dynamics of spatial-temporal orienting of attention in younger and older adults

Simone G. Heideman, Gustavo Rohenkohl, Joshua J. Chauvin, Clare E. Palmer, Freek van Ede & Anna C. Nobre

Oxford Centre for Human Brain Activity, Wellcome Centre for Integrative Neuroimaging, Department of Psychiatry, University of Oxford, UK

Content

Figures S1 – S5

Tables S1 – S4

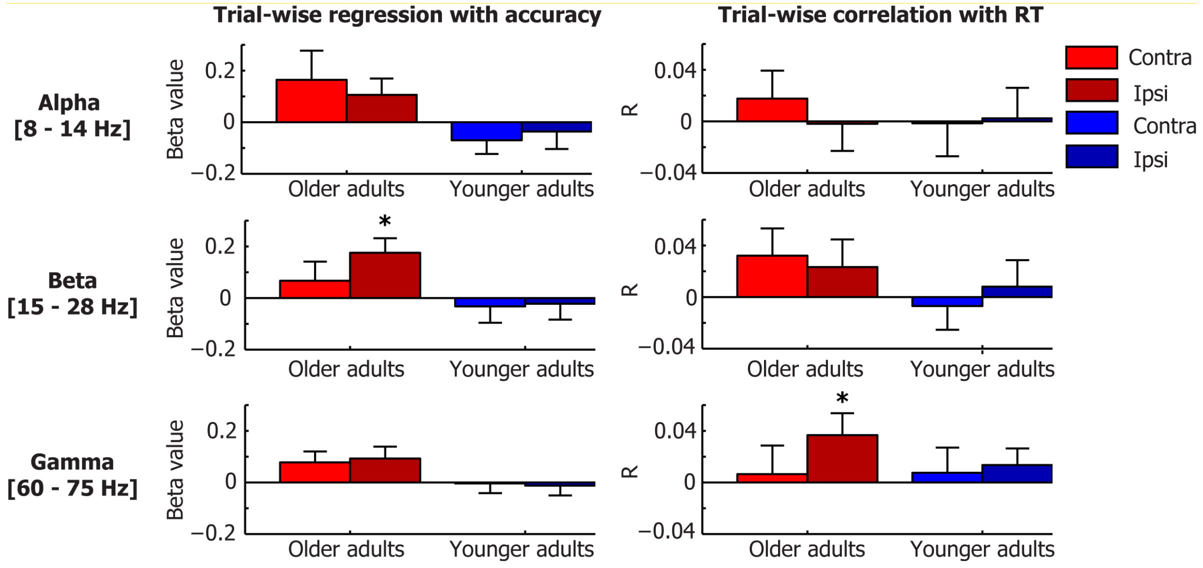

Figure S1. Trial-wise regression of MEG power with accuracy and trial-wise correlation of MEG power with RT. Trial-wise regression of accuracy (correct or incorrect; shown on the left) and correlation with RT (shown on the right) with MEG power in alpha (8-14 Hz), beta (15 – 28 Hz) and gamma (60 -75 Hz) for a time window between 300 and 650 ms following valid short cue onset. Asterisks indicate statistically significant effects (t-test against zero, uncorrected for multiple comparisons).

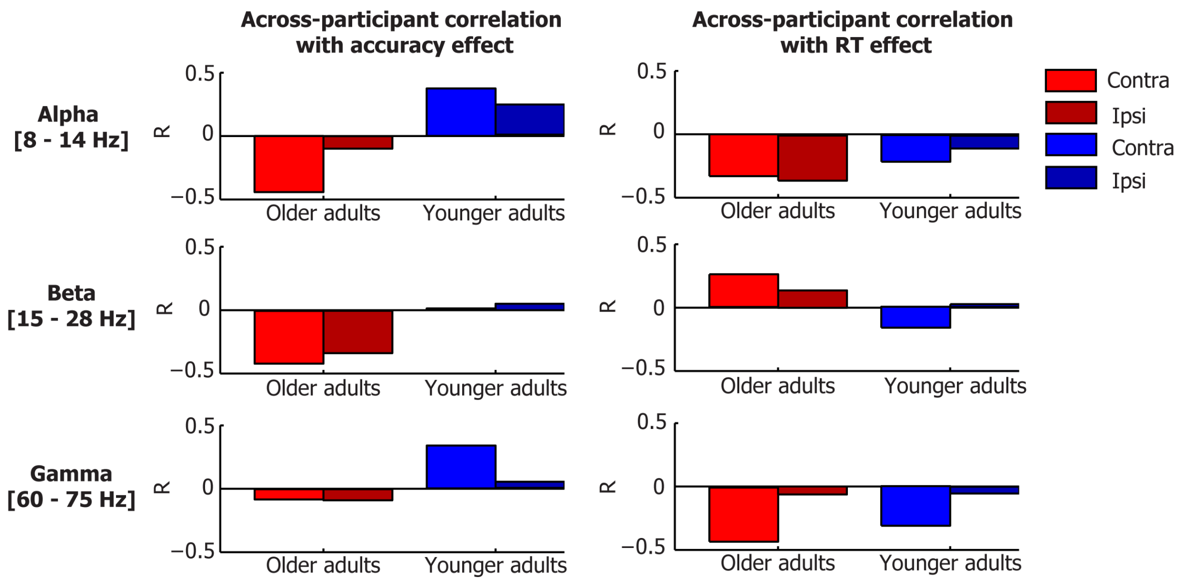

Figure S2. Across-participant correlation of MEG power with behavioural performance for accuracy and RT. Correlation with the behavioural validity effect (performance for short valid cue trials minus long invalid cue trials) for accuracy (shown on the left) and RT (shown on the right) MEG power in alpha (8-14 Hz), beta (15 – 28 Hz) and gamma (60 -75 Hz) for a time window between 300 and 650 ms following valid short cue onset. None of these correlations were statistically significant.

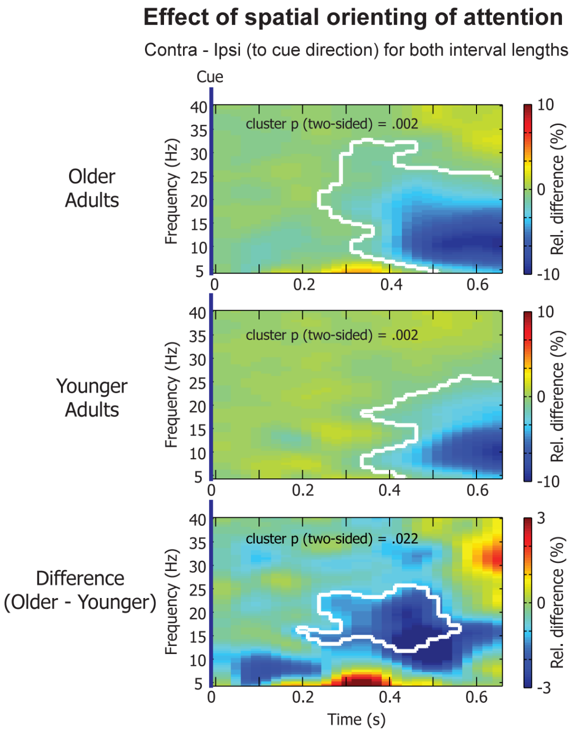

Figure S3. MEG results for the effect of spatial orienting of attention, tested for the window without possible influence of target presentation (0 – 650 ms after cue onset) only. Time-frequency representation (TFR) plots for older adults, younger adults and the difference between both groups (older-minus-younger), for contralateral ROI channels minus ipsilateral ROI channels (to the direction of the cue), averaged over both cue-target interval lengths. The colour scale indicates a relative increase or decrease. Significant clusters are outlined in white.

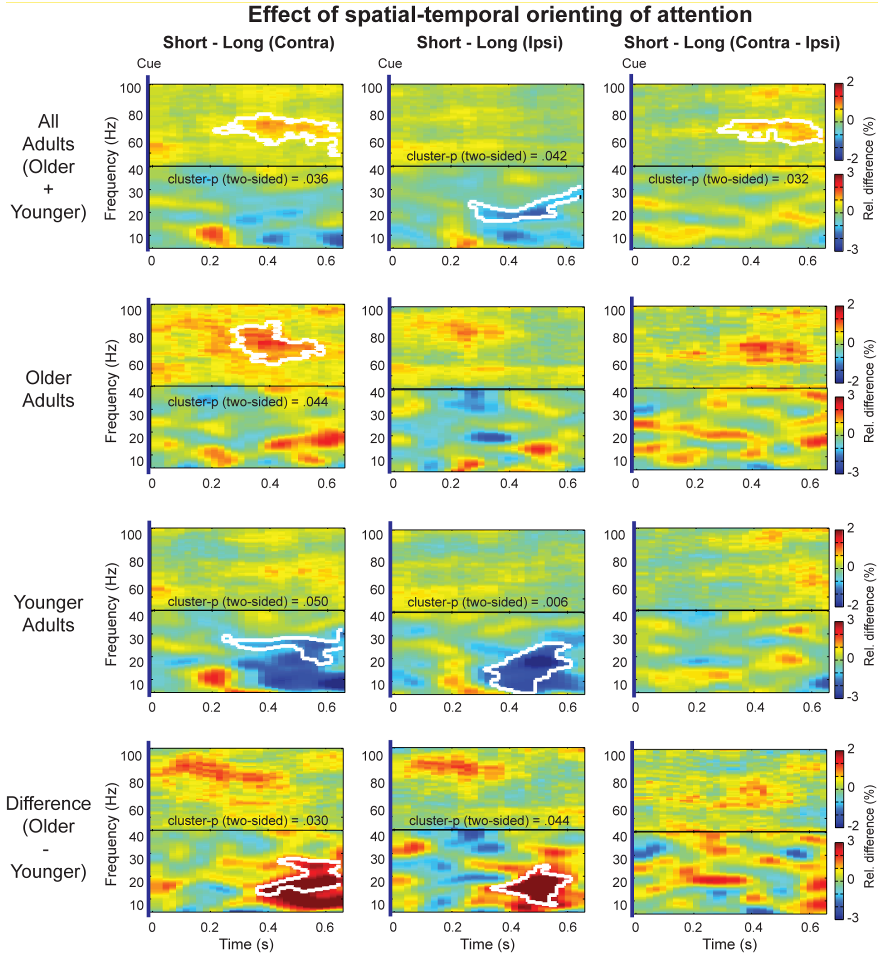

Figure S4. MEG results for the effect of spatial-temporal orienting of attention, tested for the window without possible influence of target presentation (0 – 650 ms after cue onset) only. Time-frequency representation (TFR) plots showing the short-minus-long cue contrast for all participants (older-plus-younger), older adults, younger adults and the difference between groups (older-minus-younger), separately for ROI channels contralateral (first column) and ipsilateral (second column) to the cue direction and for the contra-minus-ipsi contrast (third column). The colour scale indicates a relative increase or decrease. Significant clusters after nonparametric cluster-based permutation testing are outlined in white.

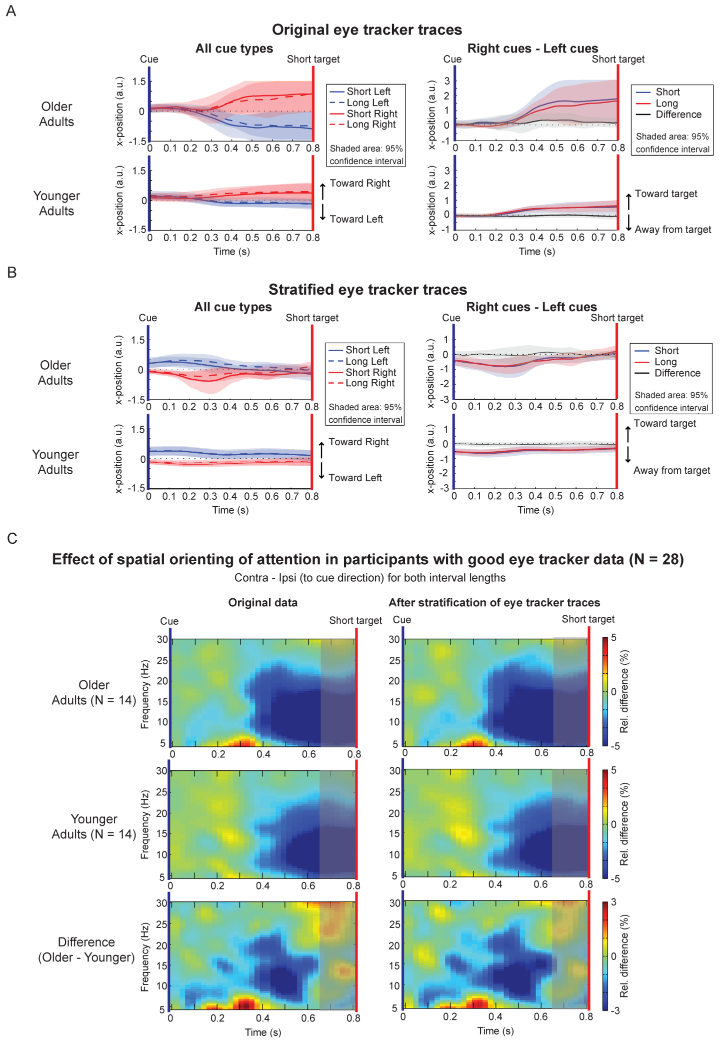

Figure S5. Effect of spatial orienting of attention before and after stratification of eye-tracking data. (A) Original eye tracking traces for the 14 older (top) and 14 younger (bottom) adults with usable eye tracking data (identical to Figure 4A). The left side shows data for all cue types. The y-axis shows the x-position of the eye tracker, with 0 being completely centre. The right side shows data for the size of the difference in x position between left and right cues, for both the short and the long interval. (B) Reverted eye tracking traces for the older and younger adults with usable eye tracking data. The most left or right biased trials were removed in an iterative fashion, until participant averages of short and long left cue trials between 200 and 800 ms pointed to the right (i.e. were above zero), and averages of short and long right cue trials pointed to the left (i.e. were below zero). (C) Time-frequency representation (TFR) plots for the 28 participants with usable eye tracking data, before (left) and after (right) stratification of eye tracking traces. Data are shown for older adults, younger adults and the difference between both groups, for contralateral ROI channels minus ipsilateral ROI channels (to the direction of the cue), averaged over both cue-target interval lengths, for the short interval (0 - 800 ms). The colour scale indicates a relative increase or decrease.

Table S1

Trial-wise regression of power between 0.3 and 0.65 s after cue onset with accuracy in valid short cue trials. Uncorrected for multiple comparisons.

	Contra			Ipsi		
	df	t	Sig. (2-tailed)	df	t	Sig. (2-tailed)
Alpha						
Older adults	15	1.46	.17	15	1.68	.11
Younger adults	19	-1.32	.20	19	-0.54	.60
Beta						
Older adults	15	0.90	.38	15	3.12	.007
Younger adults	19	-0.51	.61	19	-0.36	.72
Gamma						
Older adults	15	1.84	.09	15	2.01	.06
Younger adults	19	-0.10	.92	19	-0.32	.76

Table S2

Trial-wise correlation of power between 0.3 and 0.65 s after cue onset with RT in valid short cue trials. Uncorrected for multiple comparisons.

	Contra			Ipsi		
	df	t	Sig. (2-tailed)	df	t	Sig. (2-tailed)
Alpha						
Older adults	15	0.82	.43	15	-0.10	.93
Younger adults	19	-0.06	.95	19	0.10	.92
Beta						
Older adults	15	1.52	.15	15	1.09	.29
Younger adults	19	-0.38	.71	19	0.39	.70
Gamma						
Older adults	15	0.29	.77	15	2.15	.049
Younger adults	19	0.38	.71	19	1.05	.31

Table S3

Across-participant correlation of power in short vs. long cue trials (rel. difference) between 0.3 and 0.65 s after cue onset with the validity effect for accuracy in short cue trials (percentage correct in valid short cue minus invalid long cue trials). Uncorrected for multiple comparisons.

	Contra			Ipsi		
	df	R	Sig. (2-tailed)	df	R	Sig. (2-tailed)
Alpha						
Older adults	15	-0.44	.09	15	-0.10	.71
Younger adults	19	0.37	.10	19	0.24	.31
Beta						
Older adults	15	-0.42	.11	15	-0.33	.21
Younger adults	19	0.01	.96	19	0.05	.84
Gamma						
Older adults	15	-0.08	.77	15	-0.09	.75
Younger adults	19	0.34	.15	19	0.05	.84

Table S4

Across-participant correlation of power in short vs. long cue trials (rel. difference) between 0.3 and 0.65 s after cue onset with the validity effect for RT in short cue trials (RT in valid short cue trials minus invalid long cue trials). Uncorrected for multiple comparisons.

	Contra			Ipsi		
	n	R	Sig. (2-tailed)	df	R	Sig. (2-tailed)
Alpha						
Older adults	15	-0.33	.21	15	-0.35	.18
Younger adults	19	-0.21	.37	19	-0.10	.67
Beta						
Older adults	15	0.26	.33	15	0.14	.61
Younger adults	19	-0.17	.49	19	0.02	.92
Gamma						
Older adults	15	-0.43	.10	15	-0.06	.82
Younger adults	19	-0.31	.18	19	-0.05	.82
